# Artesunate Induces Apoptosis of Bladder Cancer Cells by miR-16 Regulation of COX-2 Expression

**DOI:** 10.3390/ijms150814298

**Published:** 2014-08-15

**Authors:** Wei Zuo, Zhen-Zhong Wang, Jun Xue

**Affiliations:** Department of Urinary Surgery, the Second Affiliated Hospital of Nanjing Medical University, Nanjing 210011, China; E-Mails: zhenzhongwang001@gmail.com (Z.-Z.W.); junxue456@gmail.com (J.X.)

**Keywords:** Artesunate, bladder cancer, apoptosis, miR-16, COX-2

## Abstract

Bladder cancer is the most common malignant tumor of the urinary tract and remains one of the major causes of cancer death worldwide. In this study, we investigated the effect and mechanism of Artesunate (ART), a traditional Chinese medicine, on inducing apoptosis of human bladder cancer cells. *In vivo* antitumor activity was investigated in bladder cancer in rat by subcutaneous injection of different concentration of ART. The effect of ART on growth inhibition and apoptosis of bladder cancer cells was evaluated using dimethylthiazoly-2,5-diphenyltetrazolium bromide (MTT) assay and flow cytometry analysis, respectively. Cyclooxygenase-2 (COX-2) and miR-16 expression levels were determined with real-time PCR. The concentrations of prostaglandin E2 (PGE2) in the supernatants of bladder cancer cells were measured with an ELISA kit. The miR-16 inhibitor or mimic were transfected into cells to up- or down-regulate miR-16 expression. ART efficiently inhibited orthotopic tumor growth in the bladder cancer rat, which is accompanied with an increase of miR-16 expression and a decrease of COX-2 expression. *In vitro*, ART could induce cytotoxicity and apoptosis in bladder cancer cells, but presented a much lighter toxicity effect against normal human urothelial cells. ART significantly increased miR-16 expression and decreased the expression of COX-2 and the production of PGE2. More importantly, down-regulation of miR-16 expression could reverse the effect of ART on apoptosis and COX-2 expression in bladder cells. Moreover, exogenous PGE2 could inhibit apoptosis of bladder cancer cells treated with ART. In conclusion, ART can elicit an anti-tumor effect against bladder cancer by up-regulation of miR-16 expression, which resulted in the decrease of COX-2 expression and PGE2 production. Hence, ART might be an effective drug for the treatment of bladder cancer.

## 1. Introduction

Bladder cancer is the most common malignant tumor of the urinary tract and remains one of the major causes of cancer death worldwide [1]. The traditional strategies only modestly improve response and the 5-year survival rate in patients with invasive and metastatic bladder cancer is still very low [2]. Although chemotherapy is regarded as an effective approach in the prevention of human cancer death, side effects limit the use of this treatment [3]. As a result, it is urgent to find effective drugs that prolong survival and improve quality of life, without damaging normal cells. Meanwhile, improving our understanding of the molecular events involved in the progression of bladder cancer is beneficial for the identification of potential molecular targets for new therapeutic strategies.

MicroRNAs (miRNAs) are a class of regulatory noncoding RNAs that bind to a target site in the 3'-UTR of target mRNAs [4] and there is increasing evidence that altered microRNA expression contributes to carcinogenesis [5]. Among these microRNAs, miR-16 could act as tumor suppressors in different human tumors [6]. Previous studies have demonstrated that miR-16 could bind to the 3'-UTR region of cyclooxygenase-2 (COX-2) leading to apoptosis and growth inhibition of human hepatoma cell lines [7]. Jiang *et al.* demonstrated that miR-16 expression was significantly decreased in bladder cancer tissues compared with adjacent noncancerous bladder tissues, and that over-expression of miR-16 inhibited proliferation of bladder cancer cell lines [8]. Therefore, miR-16 could be a novel therapeutic target for the treatment of bladder cancer.

COX-2, an inducible isoform of COX, plays an important role in carcinogenesis [9]. It has been reported that COX-2 expression levels are up-regulated in bladder cancers cells, which are positively associated with an increased disease stage and with reduced patient survival [10,11]. Up-regulation of COX-2 expression is implicated in stimulation of cancer cell growth and invasion and induction of bladder cancers cell apoptosis [12]. As a result, COX-2 is a promising target and selective COX-2 inhibitors have been evaluated as chemopreventive agents for treatment of bladder cancers [13]. However, the cardiovascular toxicity of COX-2 inhibitors has limited the application of this class of agents [14].

Artesunate (ART), a soluble derivative of artemisinin isolated from decocyions of traditional Chinese medicine *Artemisia annua* L. (qinghao, sweet wormword), has been widely used for malaria treatment with low toxicity to humans [15]. In recent years, there is increasing evidence that ART has anti-cancer capability [16]; ART has been shown to have a profound cytotoxic action against several tumors, such as Kaposi’s sarcoma, hepatocellular carcinoma, non-small cell lung cancer and cervical cancer [17,18,19,20]. However, whether ART can inhibit the growth of bladder cancer has not yet been reported. Therefore, in the present study, we aimed to investigate the anti-proliferative properties of ART in bladder cancer and to assess possible mechanisms and factors involved in this effect. Our data demonstrates the finding that miR-16 inhibits COX-2 expression leading to ART-induced apoptosis of bladder cancer cells.

## 2. Results and Discussion

### 2.1. Results

#### 2.1.1. Artesunate (ART) Inhibited Tumor Growth in the Bladder Cancer Rat

**Figure 1:**
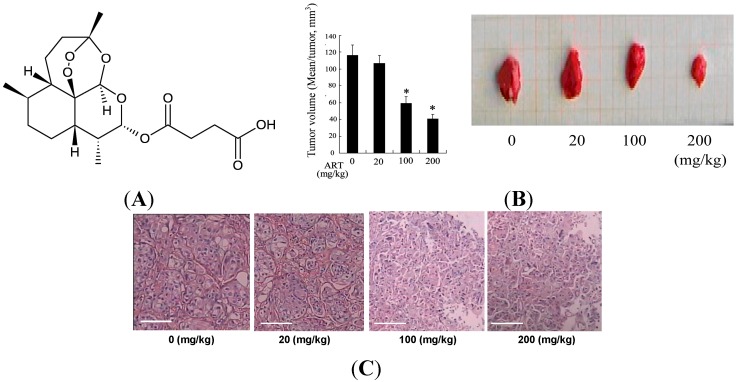
Chemical structure of Artesunate (ART) and ART inhibited tumor growth in the bladder cancer rat. Chemical structure of ART (**A**); Rats were given 0.05% of *N*-butyl-*N*-(4-hydroxybutyl) nitrosamine (BBN) in drinking water. Ten weeks later, the rats were treated with ART (0, 20, 100, 200 mg/kg) by subcutaneous injection. Ten rats were used for each treatment group. Ten weeks after treatment with ART, all rats were sacrificed and the tumor size was measured (**B**); Hematoxylin and Eosin (HE) stained cancer tissues showed ART treatment could reduce malignant lesions in a dose-dependent manner. Scale bar is equal to 20 μm (**C**); * *p* < 0.05, indicate significant differences from those treated with 0 mg/kg ART.

No relevant changes were obtained between the groups during the study concerning body weight and beverage consumption (data not shown). To evaluate the feasibility of ART therapy for bladder cancer, the efficacy of ART in inhibiting tumor growth was measured in the bladder cancer rat. In group 1, the percentage of rats with bladder cancer was 70.0% (7 in 10), with a mean of 1.1 ± 0.4 tumors per rat with tumors. A similar profile was found in groups 2, 3 and 4. However, The sizes of tumors were significantly decreased after treatment with ART (20, 100, 200 mg/kg) in a dose-dependent manner (Figure 1A).

In the group 1, there was evident malignant transformation including aggressive bladder cancers of squamous cell phenotypes. ART treatment could reduce malignant lesions. Rats in groups 3 and 4 showed no infiltrative bladder cancers and had a lower incidence of high grade tumors than those in group 1 (Figure 1B).

#### 2.1.2. ART Significantly Increased miR-16 and Decreased Cyclooxygenase-2 (COX-2) Expression in Tumors

**Figure 2:**
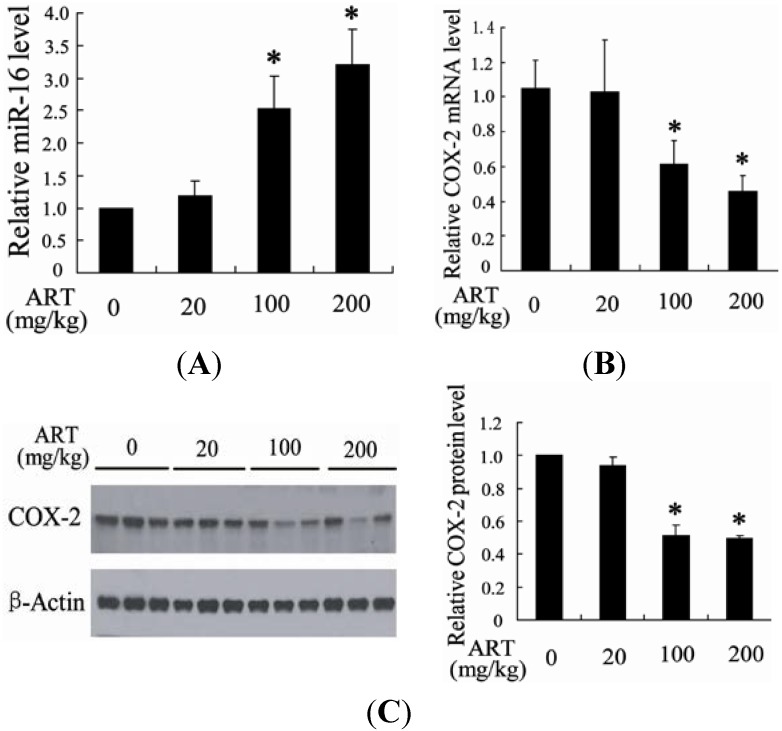
ART significantly increased miR-16 and decreased COX-2 expression in tumors. The level of miR-16 and COX-2 mRNA level in tumors from the cervical cancer rat after treatment with ART was detected (**A**,**B**); The protein level of COX-2 was measured in tumors from the cervical cancer rat after treatment with ART. Fold changes of COX-2 protein levels were also determined (**C**). * *p* < 0.05, indicates significant differences from those treated with 0 mg/kg ART.

To explore the molecular mechanism of ART inhibition of tumor growth, we measured the level of miR-16 and COX-2 in the *N*-butyl-*N*-(4-hydroxybutyl) nitrosamine (BBN)-induced tumor tissue. Compared with the rats treated with solvent control, the level of miR-16 in tumor tissue was significantly increased after treatment with ART (Figure 2A). However, the expression of COX-2 was dose-dependently decreased in BBN-induced bladder cancer rats treated with ART (Figure 2B,C).

#### 2.1.3. ART Induced Cytotoxicity and Apoptosis in Bladder Cancer Cells

**Figure 3:**
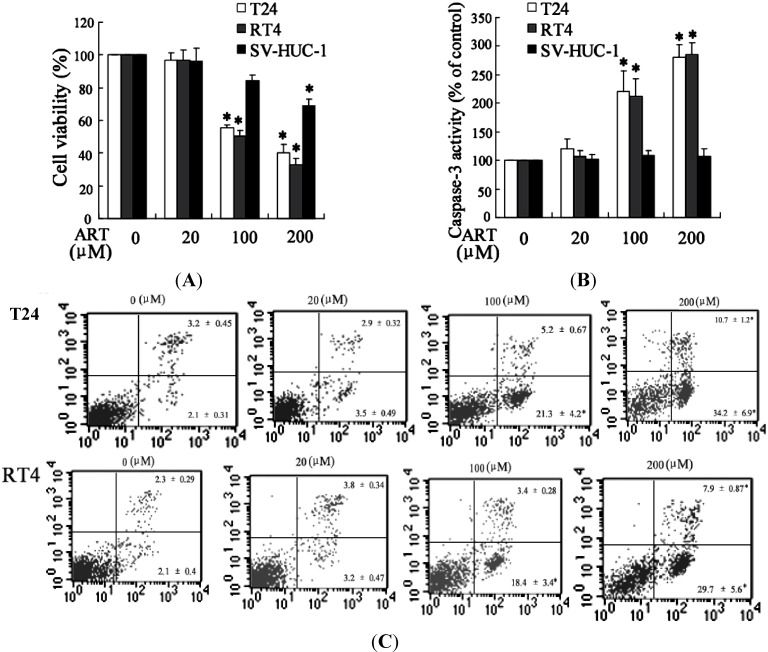
ART induced cytotoxicity and apoptosis in bladder cancer cells. The cell viability and caspase-3 activity were determined in T24, RT4 and SV-HUC-1 cells exposed to various concentrations of ART for 48 h (**A**,**B**); The apoptosis of T24 and RT4 cells were treated with different concentrations of ART for 48 h and then measured with flow cytometry (**C**). * *p* < 0.05, indicate significant differences from the control groups.

It has been reported that ART could inhibit growth and induce apoptosis of cancer cells [21]. We detected the inhibitory effects of ART on the growth of bladder cancer cells and normal human urothelial cells using dimethylthiazoly-2,5-diphenyltetrazolium bromide (MTT) assays. ART treatment significantly decreased the growth of T24 and RT4 cells in a dose-dependent manner (Figure 3A). For instance, when T24 cells were treated with 20, 100 and 200 μM of ART for 48 h, the inhibition rate of cell viability was 3.35%, 44.48% and 60.06%, respectively. And the IC_50_ value at 48 h was 129.7 μM. The similar results were obtained in RT4 cells after treatment with ART and the IC_50_ value at 48 h was 103.2 μM. ART exerts weak cytotoxicity effects on normal human urothelial cells. And the IC_50_ value at 48 h was 1149.6 μM for SV-HUC-1 cells after treatment with ART.

Activation of caspases plays an important role in the execution of apoptosis [22]. In order to determine whether ART can induce apoptosis of bladder cancer cells, we evaluated caspase-3 activity. As shown in Figure 3B, ART could significantly enhance caspase-3 activity both in T24 and RT4 cells, but could not increase that in SV-HUC-1 cells. Flow cytometry analysis further confirmed that ART could induce apoptosis of bladder cancer cells (Figure 3C).

#### 2.1.4. Down-Regulation of miR-16 Can Reverse the Effect of ART on Apoptosis of Bladder Cancer Cells

**Figure 4:**
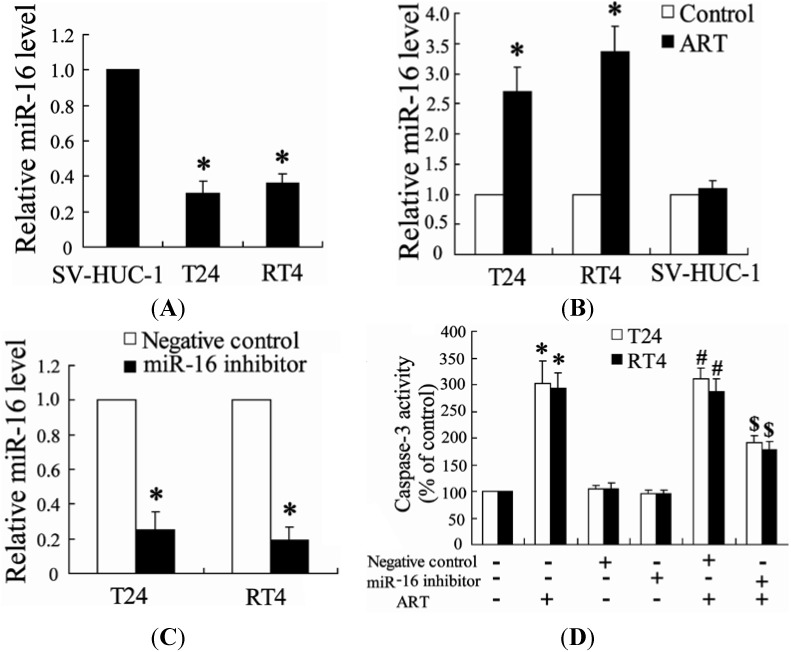
Down-regulation of miR-16 can reverse the effect of ART on apoptosis of bladder cancer cells. The level of miR-16 was measured in T24, RT4 and SV-HUC-1 cells. * *p* < 0.05, compared to SV-HUC-1 cells (**A**); The expression of miR-16 was detected in T24, RT4 and SV-HUC-1 cells treated with ART for 24 h. * *p* < 0.05, compared to vehicle (**B**); After transfection with miR-16 inhibitor for 24 h, T24 and RT4 cells were added with ART for an additional 24 h, and then the level of miR-16 was detected. * *p* < 0.05, compared to negative control (**C**); After transfection with miR-16 inhibitor for 24 h, T24 and RT4 cells were added with ART for an additional 48 h, and then caspase-3 activity was measured. * *p* < 0.05, compared to vehicle controls group. # *p* < 0.05, compared to negative control. $ *p* < 0.05, compared to negative control +ART (100 μM) treated group (**D**).

To identify whether ART could affect miR-16 expression levels in bladder cancer cells, we performed real-time PCR to detect levels of miR-16 expression. As shown in Figure 4A, the level of miR-16 was lower in bladder cancer cells (T24 and RT4 cells) than that in normal human urothelial cells (SV-HUC-1 cells). ART treatment significantly increased the expression of miR-16 in T24 and RT4 cells but did not change that in SV-HUC-1 cells (Figure 4B).

In order to assess the role of miR-16 in the effect of ART on apoptosis of bladder cancer cells, we added ART on cells after transfection with a miR-16 inhibitor. As shown in Figure 4C, the miR-16 inhibitor can significantly decrease the expression of miR-16 in T24 and RT4 cells. Interestingly, ART alone can increase the caspase-3 level, but miR-16 inhibitor with ART causes a decrease in the caspase-3 level (Figure 4D).

#### 2.1.5. ART Decreases COX-2 Expression and Prostaglandin E2 (PGE2) Production in Bladder Cancer Cells

**Figure 5:**
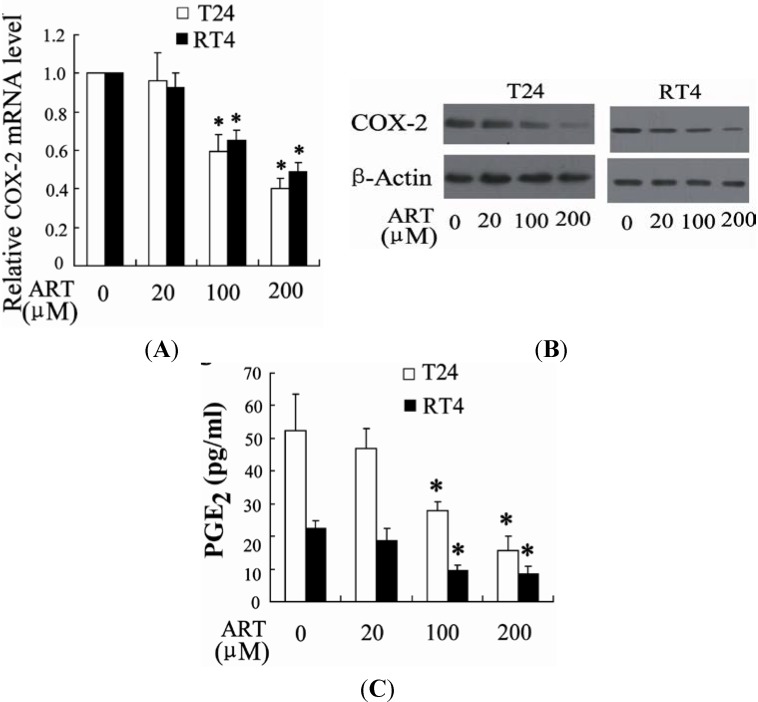
ART decreases COX-2 expression and prostaglandin E2 (PGE2) production in bladder cancer cells. T24 and RT4 cells were treated with different concentrations of ART, followed by real-time PCR (**A**) and Western blot analysis (**B**); The concentrations of PGE2 were detected in the supernatants of T24 and RT4 cells treated with different concentration of ART (**C**). * *p* < 0.05, compared to vehicle.

It has been reported that COX-2 was involved in growth inhibition and apoptosis of bladder cancer cells [23]. We also examined the effect of ART on the expression of COX-2 using real time PCR and western blot. Treatment of T24 and RT4 cells with ART for 24 h led to a dose-dependent decrease in the mRNA level of COX-2 (Figure 5A). Furthermore, ART decreased COX-2 protein expression as shown in Figure 5B. A significant decrease in release of prostaglandin E2 (PGE2) in response to ART treatment was observed in T24 and RT4 cells (Figure 5C).

#### 2.1.6. miR-16 Is Involved in ART Regulation of COX-2 Expression in Bladder Cancer Cells

**Figure 6:**
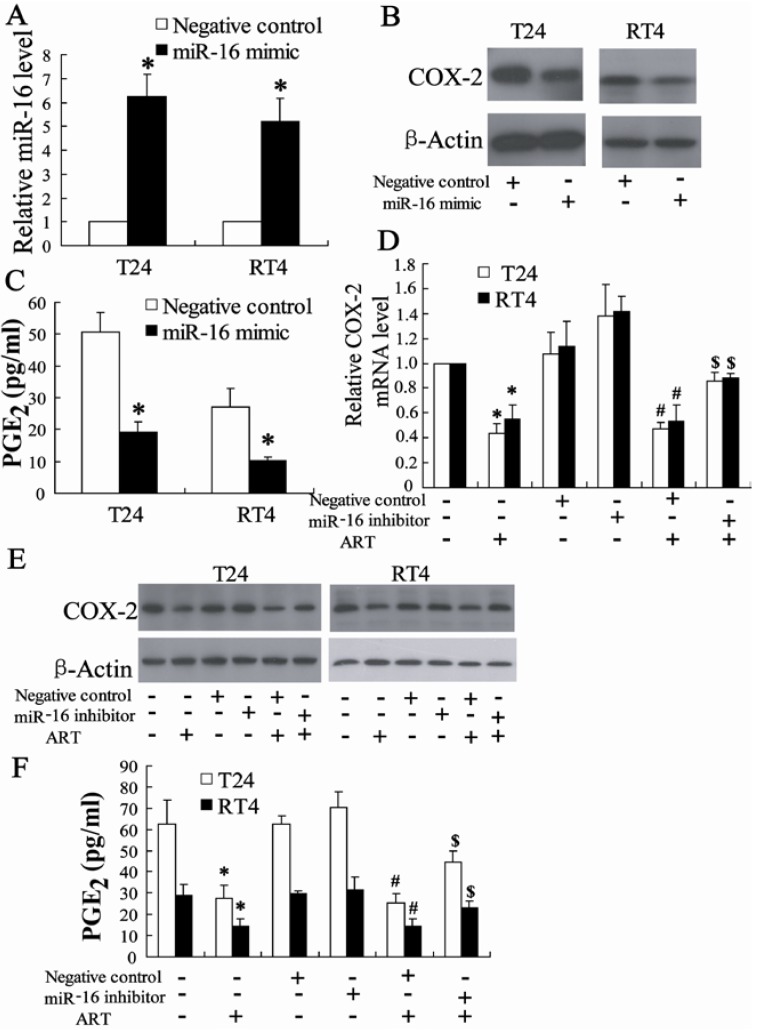
miR-16 is involved in ART regulation of COX-2 expression in bladder cancer cells. The level of miR-16 was detected in T24 and RT4 cells transfected with miR-16 mimic for 24 h. * *p* < 0.05, compared to negative control (**A**); COX-2 protein expression (**B**) in T24 and RT4 cells and PGE2 levels (**C**) in the supernatants of T24 and RT4 cells transfected with miR-16 mimic for 24 h. * *p* < 0.05, compared to negative control; After transfection with miR-16 inhibitor for 24 h, T24 and RT4 cells were added with ART for an additional 24 h, and then COX-2 mRNA and protein level was measured (**D**,**E**); After transfected with miR-16 inhibitor for 24 h, T24 and RT4 cells were added with ART for an additional 24 h, and then PGE2 levels in the supernatants were determined (**F**). * *p* < 0.05, compared to vehicle controls group. # *p* < 0.05, compared to negative control. $ *p* < 0.05, compared to negative control +ART (100 μM) treated group.

We first investigated whether miR-16 could regulate COX-2 expression and transfected miR-16 mimic into T24 and RT4 cells. The result of real-time PCR revealed that miR-16 mimic could significantly increase the expression of miR-16 in T24 and RT4 cells (Figure 6A). Furthermore, miR-16 mimic decreased the expression of COX-2 and the concentration of PGE2 as shown in Figure 6B,C.

Next, we explored the role of miR-16 in the effect of ART on the regulation of COX-2 expression; ART was added to cells after transfection with the miR-16 inhibitor. As shown in Figure 6, miR-16 inhibitor could increase COX-2 expression and PGE2 levels in bladder cancer treated with ART.

#### 2.1.7. Exogenous PGE2 Can Reverse the Effect of ART on Inhibition of Bladder Cancer Cell Growth

**Figure 7:**
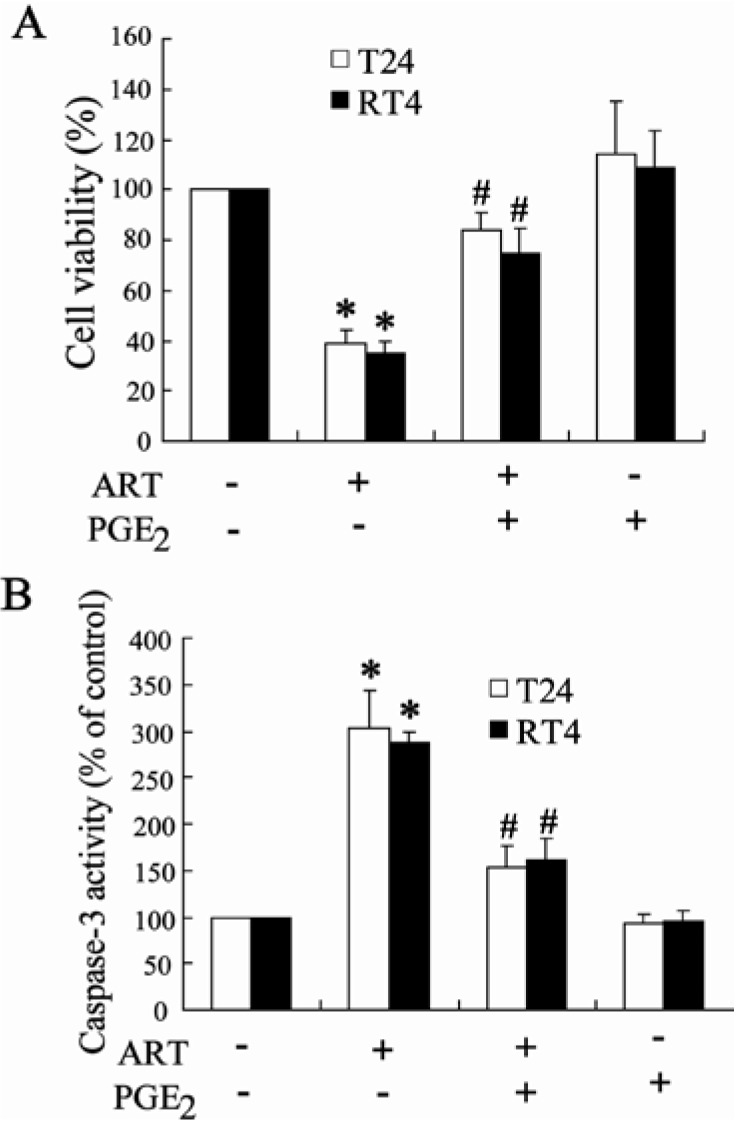
Exogenous PGE2 can reverse the effect of ART on inhibition of bladder cancer cells growth. After pretreated with exogenous PGE2 (10 μM) for 2 h, T24 and RT4 cells were added with ART (100 μM) for an additional 48 h, and then cell viability and caspase-3 activity were determined (**A**,**B**). * *p* < 0.05, compared to vehicle controls group. # *p* < 0.05, compared to only ART (100 μM) treated group.

To identify whether ART inhibits growth and induces apoptosis in a COX-2-dependent pathway, we used exogenous PGE2 to treat bladder cancer cells. As shown in Figure 7A, pretreatment of PGE2 significantly increased growth of cancer cells exposed to ART. Meanwhile, PGE2 can reverse the effect of ART on apoptosis of T24 and RT4 cells (Figure 7).

### 2.2. Discussion

ART, as a potential cancer chemoprevention agent, has a potential anti-cancer effect on several kinds of tumors such as Kaposi’s sarcoma, hepatocellular carcinoma, and non-small cell lung cancer [17,18,19,20] and ART is recognized as a safe compound to treat malaria [24] Our experimental design provides a useful model for studying the effect and molecular mechanism of ART on bladder cancer and to our knowledge, this is the first time the therapeutic effect of ART on bladder cancer has been reported. There was no difference in weight among rats in each group (data not shown), which indicated that ART could inhibit tumor growth in the bladder cancer rat without any side effects on weight maintenance. When they were tested on T24 and RT4 cell line, the IC_50_ value at 48 h after treatment was 129.7 and 103.2 μM, respectively. But the IC_50_ value for SV-HUC-1 cells was very obviously higher than that for bladder cancer cells. These results indicated that ART could specifically kill bladder cancer cells without destruction to normal human urothelial cell.

The recommended daily dose of ART is 4 mg/kg and is administered for 3 days to treat malaria in humans. However, in the present study, we used 20, 100, and 200 mg/kg of ART to treat rats with bladder cancer. Moreover, Zhang *et al.* reported that ART at the dose of 50 and 100 mg/kg could inhibit the growth of cervical cancer, which resulted from inhibiting activity of regulatory T cells through PGE2 [25]. We speculate that the difference in the concentrations levels of ART necessary for malaria treatment compared to the treatment of tumors is due to ART not being attracted specifically to tumor sites. As a result, it is important in the future to find efficient drug carriers to deliver ART specifically to tumors.

It has been demonstrated that miR-16 could regulate proliferation and apoptosis in many types of cancers including bladder cancer [26]. The tumor suppressor function of miR-16 has been addressed both *in vivo* and *in vitro* [27]. As a result, miR-16 is a promising target for treatment of cancer. Here, we found that up-regulation of miR-16 expression was associated with ART inhibition of tumor growth (Figure 2 and Figure 4B). In addition, the expression of miR-16 was found to be down-regulated in bladder cancer cells in comparison with normal urothelial cells (Figure 4). These findings suggest that up-regulation of miR-16 may be a therapeutic target for ART to treat bladder cancer. In agreement with this hypothesis, down-regulation of miR-16 expression could reverse the effect of ART on apoptosis of bladder cancer cells.

**Figure 8:**
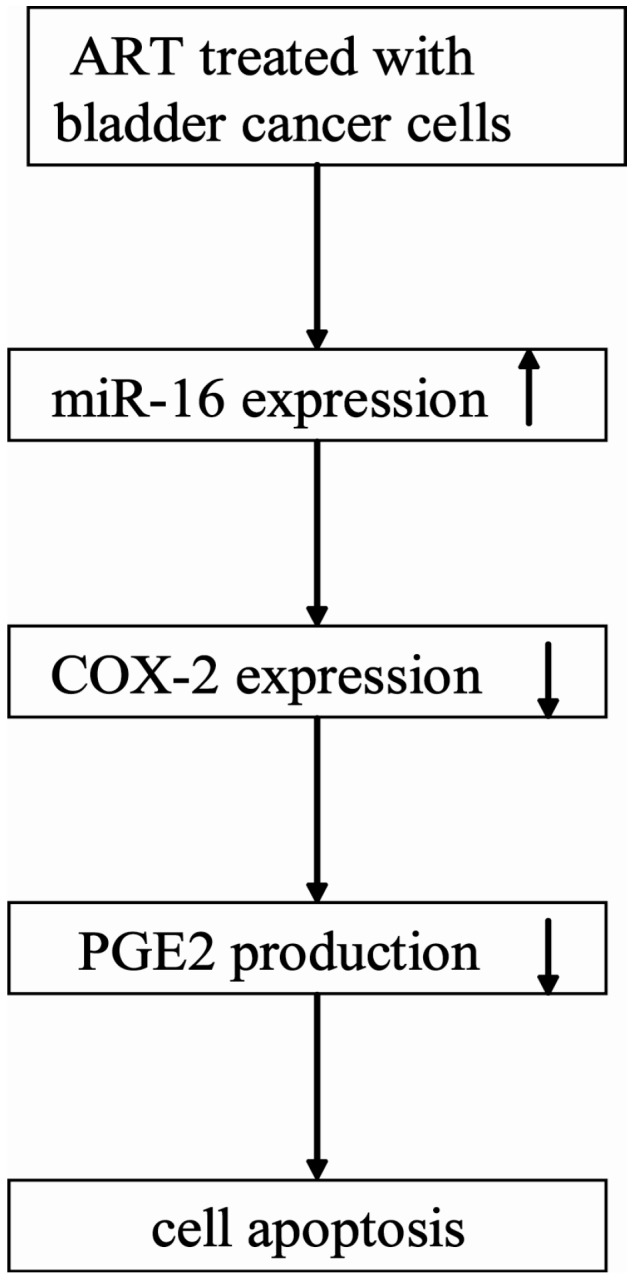
Diagram depicting the mechanism of ART induced bladder cancer cells apoptosis. The arrow of “↑” means increase and the arrow of “↓” means decrease.

Young *et al*. reported that miR-16 could bind the COX-2 3'-UTR and inhibit COX-2 expression in colorectal cancer cells [28]. We also found that over-expression of miR-16 could down-regulate COX-2 expression in bladder cancer cells (Figure 6B). Accumulating evidence suggests an important role for COX-2 in induction of cell proliferation and reduction of apoptosis of bladder cancer cells [10,11,12]. Inhibition of COX-2 activity and/or expression caused reduced cell proliferation and increased apoptosis [29]. In the present study, we found that ART inhibited the COX-2 expression of bladder cancer cells and PGE2 production in a dose-dependent manner, which could be restored by miR-16 inhibitor (Figure 6C). Furthermore, reduction in growth was observed in T24 and RT4 cells treated with ART, which was then rescued by exogenous PGE2 (Figure 7), implicating a COX-2-dependent pathway in bladder cancer cells. According to these results, we draw a conclusion that ART suppresses COX-2 expression and PGE2 production by enhancing miR-16 expression (Figure 8).

## 3. Experimental Section

### 3.1. Chemicals and Reagents

Dulbecco’s modified Eagle’s medium (DMEM) and sodium pyruvate were purchased from Gibco-BRL (Rockville, MD, USA). Fetal bovine serum (FBS) was purchased from GIBCO (Burlington, ON, USA). Dimethylthiazoly-2,5-diphenyltetrazolium bromide (MTT), ART and PGE2 were purchased from Sigma–Aldrich (St Louis, MO, USA). BBN was obtained from Tokyo Chemical Industry Co., Ltd. (Tokyo, Japan). Annexin V-enhanced green fluorescent protein/propidium iodide (V-EGFP/PI) Apoptosis Detection Kit was purchased from KeyGen (Nanjing, China). The caspase-3 activity assay kits were obtained from Beyotime (Nantong, China). Anti-COX-2 rabbit polyclonal antibody and anti-β-actin was from Santa Cruz Biotechnology (Santa Cruz, CA, USA). The Detergent Compatible (DC) Protein Assay kit was purchased from Bio-Rad Laboratories (Hercules, CA, USA). The miRNeasy Mini kit, the miScript Reverse Transcription kit and the miScript SYBR Green PCR kit were purchased from Qiagen (Hilden, Germany).

### 3.2. Cell Culture

The immortalized normal human urothelial cell line SV-HUC-1 and bladder cancer cell lines T24 and RT4 were purchased from the Cell Bank of Type Culture Collection of Chinese Academy of Sciences (Shanghai, China). These cells were cultured in DMEM supplemented with 10% FBS, 10 mM HEPES, 100 U penicillin/mL and 10 μg streptomycin/mL at 37 °C in a humidified atmosphere containing 95% air and 5% CO_2_. ART and PGE2 were dissolved in dimethyl sulfoxide (DMSO), the final concentration of DMSO in the culture medium was kept less than 0.1%. Vehicle controls were prepared for all treatments.

### 3.3. Animals

All animal studies were performed according to guidelines established by the Research Animal Care Committee of Nanjing Medical University, China (Permit Number: NJMU-ERLAUA-20120507). Male Wistar rats (grade SPF; 200g) were purchased from Shanghai Laboratory Animal Centre (Chinese Academy of Sciences, Shanghai, China). The urinary bladder cancer model was performed according to a previously reported method [30]. Briefly, rats were given 0.05% of BBN in drinking water for several weeks. Then, rats (*n* = 40) were randomly and equally divided into 4 groups 10 weeks after drinking water containing 0.05% of BBN. Group 1 rats received corn oil by subcutaneous injection, once per day. Groups 2, 3 and 4 rats were treated with 20, 100 and 200 mg/kg of ART, respectively. Ten weeks after treatment with ART, all rats were sacrificed and the tumor size was measured. Tumor volume was calculated assuming a spherical shape, with the average tumor diameter being the square root of the product of cross-sectional diameters.

### 3.4. Dimethylthiazoly-2,5-diphenyltetrazolium Bromide (MTT) Assay

Cell viability was determined using MTT [3-(4,5-dimethylthiazol-2-yl)-2,5-diphenyltetrazolium bromide assays. Briefly, the cells were seeded in 96-well dishes at 1 × 104 cells per well, and treated with different concentrations of ART for 48 h. Then each well was supplemented with 10 μL MTT and incubated for 4 h at 37 °C. The medium was then removed, and the resultant MTT formazan was solubilized in 150 μL DMSO. The optical density was read at 490 nm.

### 3.5. Flow Cytometry Analysis

Flow cytometry analysis is based on the translocation of phosphatidylserine from the inner leaﬂet of the plasma membrane to the cell surface in early apoptotic cells [31]. Briefly, cells were resuspended in a binding buffer. Next, annexin V-EGFP and PI were added and the solution was incubated at room temperature for 15 min in the dark, followed by assay on FACScan (Becton Dickinson, East Rutherford, NJ, USA). The percentage of apoptosis was computed using Cell-Quest software (Becton Dickinson).

### 3.6. Caspase-3 Activity Assays

Caspase-3 activity was analyzed using the caspase-3 activity assay kit according to the manufacturer’s protocol. Cells were lysed and total cellular protein extracts were quantified using a protein-assay kit. Next, an equal amount of total protein extract was incubated at 37 °C overnight with either Ac-IETD-pNA for caspase-3 assay. The release of pNA was estimated by determining the absorbance at 405 nm on a microplate ELISA reader (Bio-Rad Laboratories, Hercules, CA, USA). The relative activity of caspase-3 was calculated as follows: caspase-3 activity = (mean experimental absorbance/mean control absorbance) × 100 (%).

### 3.7. ELISA

Analysis of PGE2 levels in the culture medium was performed using an ELISA kit according to the manufacturer’s protocol. The corresponding cells were lysed, and the protein concentration was determined to normalize the PGE2 levels measured in the medium samples.

### 3.8. Real-Time PCR

Mature miRNAs of cultured SV-HUC-1, T24 and RT4 cells was isolated utilizing the miRNeasy Mini kit and reverse-transcribed with the miScript Reverse Transcription kit in accordance with the manufacturer’s instructions. The miR-16 level was quantified by real-time PCR using TransStart™ SYBR Green qPCR Supermix (TransGen Biotech, Beijing, China), and with U6 small nuclear RNA as an internal normalized reference. For miR-16, the primers were as follows: forward, 5'-TAGCAGCACGTAAATATTGGCG-3' and reverse, 5'-CCAGTATTGACTGTGCTGCTGA-3'. For U6, the primers were as follows: forward, 5'-GCTTGCTTCGGCAGCACATATAC-3' and reverse, 5'-TGCATGTCATCCTTGCTCAGGG-3'. The mRNA level of COX-2 was also quantified by real-time PCR. The specific primers were as follows: COX-2, 5'-CCGAGGTGTATGTATGAGTG-3' (forward) and reverse: 5'-AACTGATGCGTGAAGTGCTG-3' (reverse); β-actin, 5'-GATGAGATTGGCATGGCTTT-3' (forward); and 5'-CACCTTCACCGTTCCAGTTT-3' (reverse).

### 3.9. Western Blot Analysis

T24 and RT4 cells were lysed with ice-cold lysis buffer containing: 50 mmol/L Tris–HCl, pH 7.4; 1% NP-40; 150 mmol/L NaCl; 1 mmol/L EDTA; 1 mmol/L phenylmethylsulfonyl fluoride; and complete proteinase inhibitor mixture (one tablet per 10 mL; Roche Molecular Biochemicals, Indianapolis, IN, USA). Protein concentration in the cell lysate was quantified using the DC protein assay kit (Bio-Rad Laboratories). After protein content determination using a DC Protein Assay kit, western blot analysis was performed.

### 3.10. Transfection Procedures

miR-16 was over-expressed or by knocked down transfection with miR-16 mimic or miR-16 inhibitor. miR-16 mimics (5'-UAGCAGCACGUAAAUAUUGGCG-3'), miR-16 inhibitor (5'-CCAGUAUUAACUGUGCUGCUGA-3') and negative control (NC, 5'-CAGUACUUUUGUGUAGUACAA-3') were synthesized by RIBOBIO (Ribobio Co., Ltd., Guangzhou, China). All of the oligonucleotides were transfected at a final concentration of 100 nM. T24 and RT4 cells were transfected with miR-16 inhibitor or mimic using Lipofectamine 2000 reagent according to the manufacturer’s recommendations.

### 3.11. Statistical Analysis

Statistical analysis was performed with statistical analysis software SPSS 13.0 software (SPSS Inc., Chicago, IL, USA). Comparisons between groups were made using one-way ANOVA, followed by Student’s *t*-test. Results are presented as means ± SEM. Values of *p* < 0.05 were considered to be significant.

## 4. Conclusions

We found that a reduction in tumor cell proliferation accounted for the therapeutic effect of ART on bladder cancer. This is in line with the finding that increased miR-16 levels correlated with decreased expression of COX-2 in bladder cancer cells. We have established miR-16 was involved in ART mediating cytotoxicity and apoptosis of bladder cancer cells. Our data support the hypothesis that ART stimulates miR-16 expression, leading to the reduction of COX-2 expression levels, which resulted in a decrease of PGE2 production. These data indicate ART is an effective medicine to treat human bladder cancer.
